# Supplementary material for: Colorimetric Kit for Rapid Porcine Circovirus 2 (PCV-2) Diagnosis
Source: Pathogens. 2022 May 12;11(5):570. doi: 10.3390/pathogens11050570 (PMC9147935; doi:10.3390/pathogens11050570)
Supplement: Supplementary file 1 [file pathogens-11-00570-s001.zip › pathogens-1678469-supplementary.pdf]

Support Information

Colorimetric Kit for Rapid Porcine Circovirus 2 (PCV-2) Diagnosis

Caroline Rodrigues Basso[1]; Ana Carolina Yamakawa[1]; Taís Fukuta Cruz[1,2]; Valber Albuquerque Pedrosa[2]; Massimiliano Magro[3]; Fabio Vianello[3] João Pessoa Araújo Júnior[1,2].

[1] *Biotechnology Institute, São Paulo State University- Botucatu, SP 18607-440, Brazil.*

[2] *Chemical and Biological Sciences Department, Bioscience Institute, São Paulo State University- Botucatu, SP 18618-000, Brazil.*

[3] *Comparative Biomedicine and Food Science Department, University of Padua, 35020 Legnaro, Italy.*

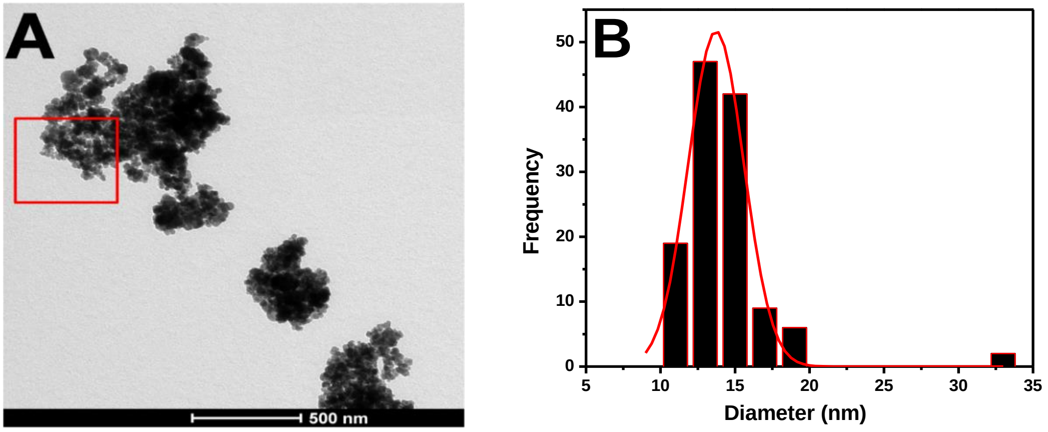

Figure S1. Surface active maghemite nanoparticles' (SAMN) featuring. **(A)** Transmission electron microscopy (TEM) image; and **(B)** histogram showing mean nanoparticle diameter of 13.72 nm.

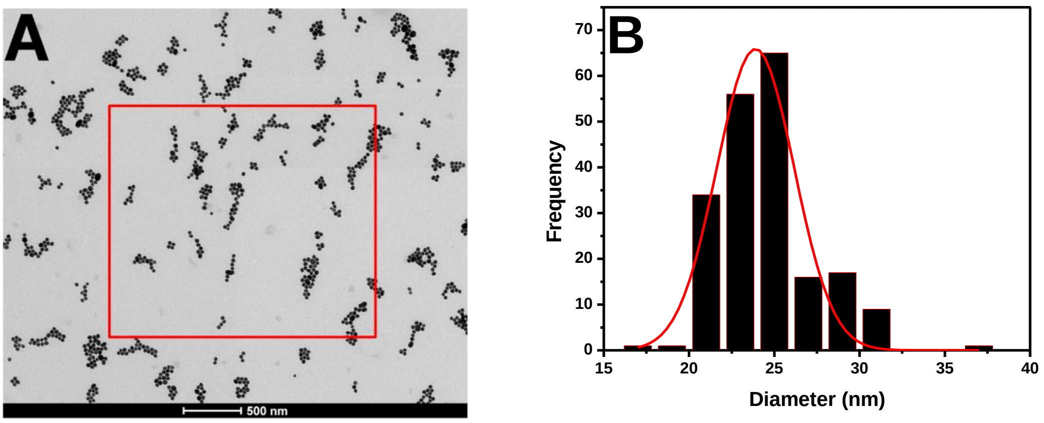

Figure S2. Gold nanoparticles' (AuNPs) featuring. **(A)** Transmission electron microscopy image; and **(B)** histogram showing mean nanoparticle diameter.

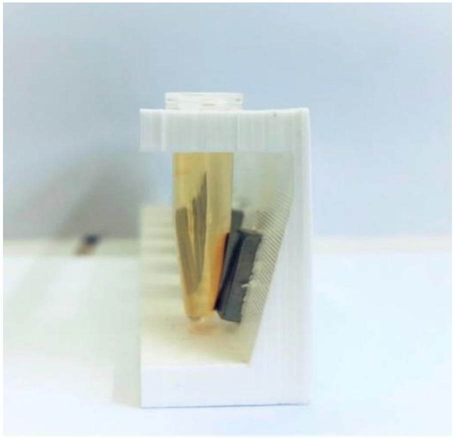

Figure S3. SAMN retrieved from water suspension upon exposure to external magnet.

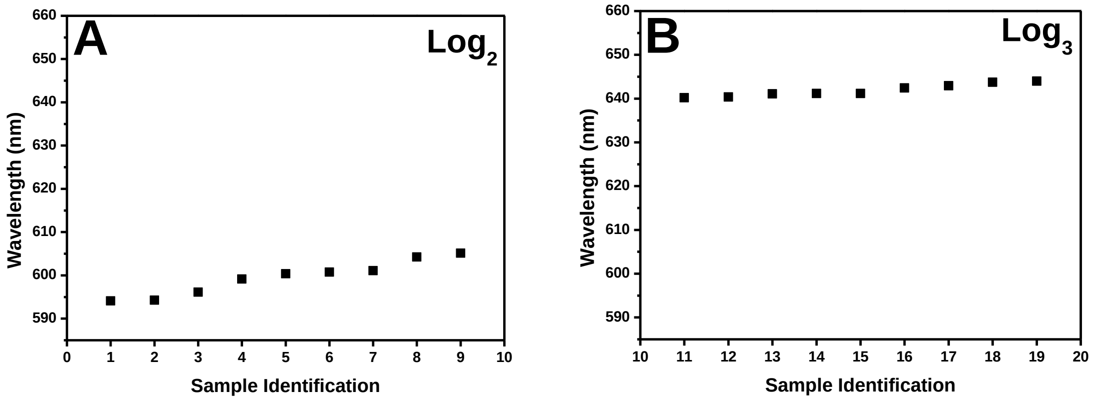

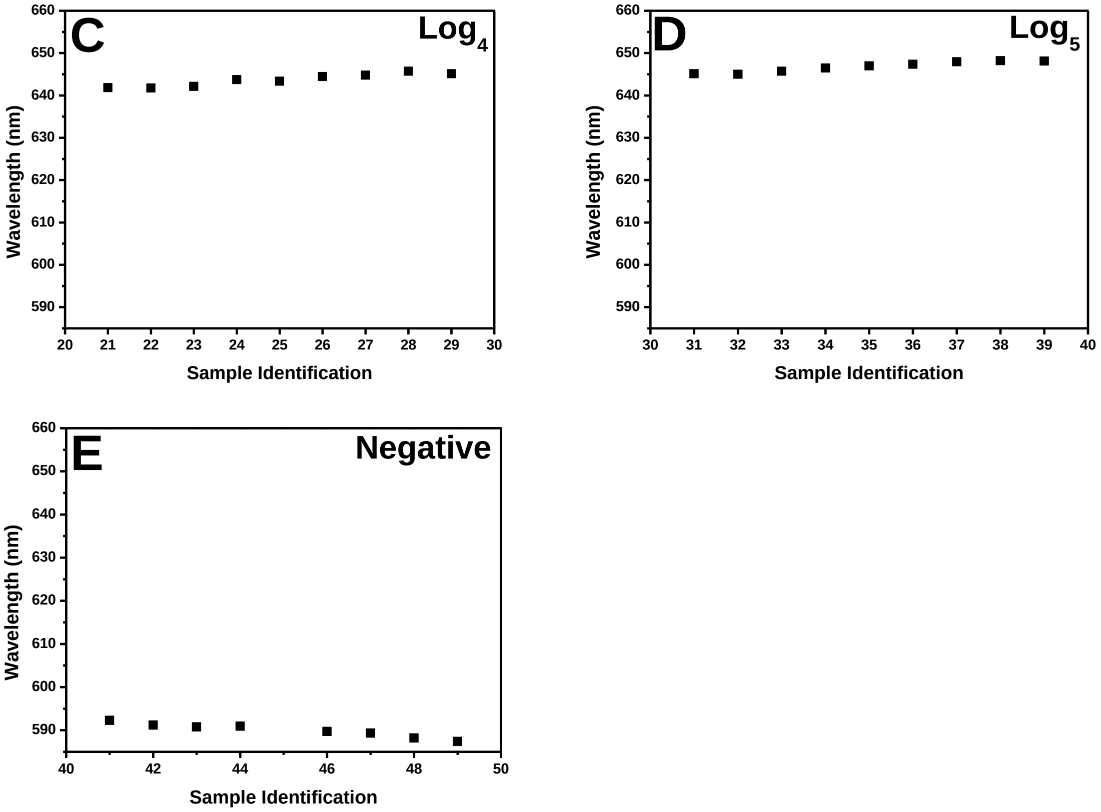

Figure S4: Spectrophotometer graphs showing the wavelengths of different viral load of samples tested. **(A)** samples with viral load of log_2, **(B)** samples with viral load of log_3, **(C)** samples with viral load of log_4, **(D)** samples with viral load of log_5 and **(E)** negative samples.

Table S1: pH values for positive samples.

**Sample Identification**	**Log_{10} (copies/μL)**	**pH**
SO10754	2.41	5.1
SO10765	2.62	5.0
SO11948	2.71	5.5
SO12166	2.63	5.0
SO12178	2.01	5.0
SO12669	2.57	5.0
SO12670	2.87	5.4
SO2G3	2.39	5.5
SO3G3	2.41	5.5
SO6G3	2.97	5.4
SO8783	3.02	5.5
SO8786	3.71	5.0
SO8791	3.42	5.0
SO8808	3.62	5.7

SO10767	3.56	5.5
SO10768	3.02	5.3
SO10774	3.72	5.1
SO10779	3.84	5.0
SO10782	3.20	5.0
SO 12659	3.15	5.0
SO8777	4.18	5.1
SO8797	4.41	5.0
SO8798	4.68	5.7
SO8803	4.49	5.5
SO8810	4.82	5.5
SO8819	4.00	5.5
SO8823	4.73	5.1
SO11974	4.94	5.7
SO5G1	4.80	5.5
SO7G1	4.19	5.5
SO8787	5.91	5.5
SO8790	5.27	5.2
SO8788	5.38	5.0
SO8799	5.10	5.0
SO8800	5.17	5.0
SO8802	5.57	5.5
SO8820	5.05	5.5
SO8795	5.95	5.7
SO12468	5.09	5.0
SO12479	5.05	5.0

Table S2: pH values for negative samples.

**Samples Identification**	**Log_{10} (copies/µL)**	**pH**
SO12671	0	5.0
SO12673	0	5.0
SO12674	0	5.0
SO12680	0	5.5
SO12681	0	5.0
SO12683	0	5.0
SO12686	0	5.5
SO12687	0	5.5
SO12688	0	5.5
SO12689	0	5.3
